# State of the Art on Vaccine Development Against Dengue Infection: Scoping Review of the Literature

**DOI:** 10.3390/idr17050117

**Published:** 2025-09-17

**Authors:** Davide Marangoni, Anna Barbiero, Michele Spinicci, Alessandro Bartoloni, Andrea Rossanese, Paolo Bonanni, Lorenzo Zammarchi

**Affiliations:** 1 Department of Experimental and Clinical Medicine, University of Florence, 50134 Florence, Italy; 2 Department of Infectious and Tropical Diseases, Careggi University Hospital, 50134 Florence, Italy; 3 Department of Infectious-Tropical Diseases and Microbiology, IRCCS “Sacro Cuore-Don Calabria”, Negrar di Valpolicella, 37024 Verona, Italy; 4 Department of Health Sciences, University of Florence, 50134 Florence, Italy

**Keywords:** dengue, vaccine, CYD-TDV, TAK-003, TV003, TV005

## Abstract

**Background:** Dengue virus infection is a significant challenge for global health, with 100 million symptomatic cases, 2.3 million DALYs and 20,000 deaths annually. Dengue vaccines must provide long-lasting immunity against all four virus serotypes, especially in dengue-naïve individuals, in order to avoid the severe manifestations of secondary infections. **Methods:** This scoping review summarizes current evidence on licensed dengue vaccines and vaccine candidates, focusing on immunogenicity, efficacy, and safety outcomes. To identify relevant trials, in October 2023 we queried ClinicalTrials.gov using the search term “dengue vaccines” to identify past and present vaccine candidates; the search was repeated in February 2025. Vaccines were categorized into licensed (CYD-TDV and TAK-003), late-stage (TV003/TV005), and early-stage candidates (TDEN, DPIV, V180, TVDV). **Results:** CYD-TDV (Dengvaxia^®^) showed moderate efficacy in large trials, with higher efficacy in seropositive than in seronegative individuals. Following commercialization, an increased hospitalization risk was discovered in the latter group. Due to these findings and impossibility of screening for prior exposure in endemic settings newer vaccines are now preferred and CYD-TDV production has recently been discontinued due to declining demand. TAK-003 (Qdenga^®^) demonstrated high efficacy against virologically confirmed dengue (VCD) and dengue-related hospitalization. This vaccine was generally well tolerated and is currently recommended by scientific societies and national authorities for travelers and by WHO for routine use in adults and children in endemic settings. TV003 and TV005, developed by NIAID, showed strong immunogenicity and efficacy in phase II trials and human challenge models. Preliminary results show that a single-dose of TV003 has an efficacy of 79.6% in seronegatives and 89.2% in seropositives against VCD at a 2-year follow-up. Both formulations elicited tetravalent responses with an acceptable safety profile. Other vaccine strategies, including TDEN (live-attenuated), DPIV (purified inactivated), V180 (subunit), and TVDV (DNA-based) are still in early-phase development and suffer from waning antibody titers and limited efficacy in naïve subjects. **Conclusions:** The development of a safe and effective vaccine remains complex due to immunologic challenges. Currently, TAK-003 is regarded as the best option for broad implementation, while TV003 and TV005 remain promising candidates due to their shorter schedule and robust immunogenicity. Further research is needed to optimize vaccine strategies in seronegative populations, immunocompromised subjects, older adults, and travelers.

## 1. Introduction

Dengue is a febrile infectious disease caused by one of four different serotypes of Dengue virus (DENV), an arbovirus transmitted by mosquitoes of the Aedes genus, mainly *Aedes aegypti* and *Aedes albopictus*. All serotypes may infect humans and are responsible for a clinical spectrum ranging from asymptomatic infection to a life-threatening syndrome characterized by shock, hemorrhage and multiorgan failure. Even though in 2024 the World Health Organization (WHO) reports more than 14 million yearly symptomatic dengue cases and 10,000 deaths [1], estimates suggest that 390 million people are infected annually worldwide, with nearly 100 million developing clinical manifestations of the disease, and a burden of disease exceeding 2.3 million disability-adjusted life-years (DALYs), 500,000 hospitalizations per year, and 20,000 deaths per year. Mortality rates are higher in children under 5 years of age and adults over 70. Up to 3.9 billion people worldwide are at risk of infection [2,3,4]; this number is already increasing [1] and expected to rise further due to climate change, as rising temperatures are predicted to alter mosquito breeding patterns and geographic distribution, potentially expanding the areas at risk [5,6]. Both *Aedes aegypti* and *Aedes albopictus* have indeed progressively extended their range into temperate regions (e.g., Southern Europe, Paraguay, Argentina) [7,8] and higher altitudes (e.g., Peruvian Andes) [9], with autochthonous dengue infections being reported with increasing frequency in these settings.

Primary infection typically presents with a fever lasting 3–7 days, accompanied by symptoms such as headache, vomiting, arthromyalgia, thrombocytopenia, leukopenia, and/or a moderate elevation of aminotransferase levels. Most individuals recover without further complications; however, particularly in children and adolescents, defervescence may be followed by a systemic vascular leak syndrome, presenting with plasma leakage, hemoconcentration, bleeding, and potentially shock [6]. Although both conditions can occur with primary dengue, most cases of severe dengue are associated with secondary heterologous infections, due to a phenomenon called antibody-dependent enhancement (ADE), consisting of the production of non-neutralizing antibodies (such as those directed against other serotypes) that facilitate the entry of other DENV serotypes into immune system cells [10]. It is worth noting that third and fourth infections are not associated with increased severity and are usually milder than the primary infection, as the immunity acquired during multiple infections grants higher levels of cross-protective and heterotypic neutralizing antibodies [10,11]. Conversely, travelers tend to experience severe dengue in 2% of cases, with no marked difference between primary and secondary infections [12,13]. These facts underscore the need for a vaccine capable of protecting young children, elderly adults and travelers.

Currently, only two vaccines are licensed for commercial use: CYD-TDV (chimeric yellow fever virus—DENV-tetravalent dengue vaccine), sold since 2016 under brand name Dengvaxia, which was developed by Sanofi Pasteur (Lyon, France) and is no more in production, and TAK-003, sold since 2022 under brand name Qdenga by Takeda Pharmaceuticals (Tokyo, Japan). Several others are in phase I, II or III of development.

## 2. Materials and Methods

**Figure 1:**
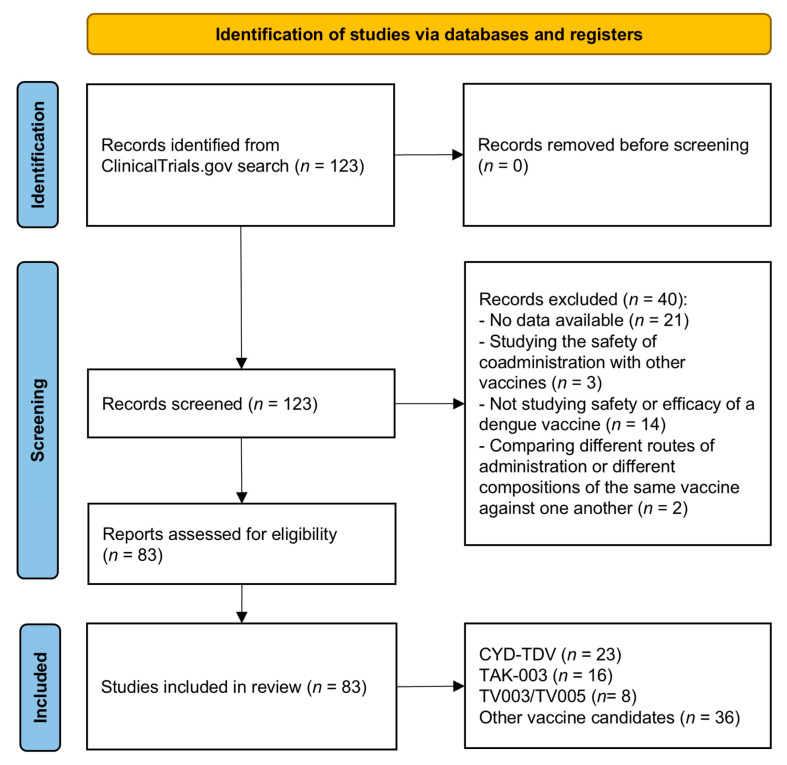
PRISMA flow diagram of the search and selection process.

In October 2023, we conducted a query on ClinicalTrials.gov using the search term “dengue vaccines” to identify past and present vaccine candidates. All retrieved trials were evaluated for inclusion based on availability of data about the safety and efficacy of the vaccine candidate. Trials with outcomes other than safety, efficacy or immunogenicity and trials with no published data were excluded. This search was repeated during the writing of this article (latest update on 9 February 2025) to ensure the inclusion of the most recent data from ongoing studies. Data from publications related to these studies were used when available; otherwise, results posted on clinicaltrials.gov were referenced. The data were then analyzed independently by two authors (D.M. and A.B.), categorized into licensed vaccines and vaccines in late-stage development (CYD-TDV, TAK-003, TV003/TV005), vaccines candidates abandoned in early-stage development (TDEN), and vaccine candidates still undergoing phase I-II trials (DPIV, V180, TVDV), and summarized with the available details about safety, immunogenicity and efficacy (overall and subgroup-specific). The selection process is highlighted in Figure 1. This scoping review was conducted following the Preferred Reporting Items for Systematic reviews and Meta-Analyses extension for Scoping Reviews (PRISMA-ScR) Checklist (available as Supplementary Material).

## 3. Results

### 3.1. CYD-TDV

CYD-TDV is a recombinant live-attenuated tetravalent vaccine based on the attenuated yellow fever YF17D vaccine strain, in which the structural membrane precursor (prM) and envelope (E) genes are substituted with the corresponding genes of each dengue serotype (specifically, Thai strain PUO-359/TVP-1140, Thai strain PUO-218, Thai strain PaH881/88 and Indonesian strain 1228 (TVP-980) [14]. Attenuation of the yellow fever backbone is achieved by inducing specific mutations through serial passage of the Asibi YF strain in cellular cultures from mouse and chicken embryonic tissue [15,16].

Pooled analysis of the three phase III studies targeting 35,146 children aged 2–16 who received either CYD-TDV or placebo at months 0, 6, and 12 in the Asia-Pacific region (CYD14 trial; 6851 participants assigned to the vaccine arm; 3424 assigned to the control arm), Latin America (CYD15 trial; 13,920 vaccine; 6949 control), and Thailand (CYD23-57 trial; 2669 vaccine; 1333 control) [17,18,19], calculated a global vaccine efficacy (VE) against asymptomatic infection of 33.5% in the first 25 months, ranging from −1.1% in seronegative participants to 41.7% in seropositive individuals [20]. The overall pooled serotype-specific efficacy against VCD in CYD14, CYD15, and CYD57 trials differed among the four serotypes: in participants aged 9–16, not considering their serostatus, it was 58.4% for DENV-1, 47.1% for DENV-2, 73.6% for DENV-3 and 83.2% for DENV-4; in participants aged 2–9, it was 46.6% for DENV-1, 33.6% for DENV-2, 62.1% for DENV-3 and 51.7% for DENV-4 [21].

The CYD-TDV vaccine was assessed again at 5 years from the first administration in a sub-cohort of the previously mentioned studies (*n* = 5622). The efficacy against symptomatic virologically confirmed dengue (VCD) was 32% at 5 years among seronegative participants and 73% among seropositive individuals. Although CYD-TDV received its first marketing authorization in 2015, data about this five-year follow-up became available only later, and revealed an increased risk associated with vaccination of seronegative individuals: the cumulative 5-year relative risk (RR) for hospitalization for VCD and severe VCD was 1.41 and 2.44 in seronegative patients and 0.21 and 0.16 in seropositive patients, respectively. Efficacy against symptomatic VCD was greater in the seropositive 9–16 age group, whereas a higher risk of hospitalization was noted in the seronegative 9–16 age group [22]. Another analysis of the Asian-Pacific, Thai and Latin American cohorts (*n* = 33,904) reported an overall RR for hospitalization due to any serotype of 0.511 over 4 years, ranging from 0.292 in seropositive patients to 1.327 in seronegative patients. While CYD-TDV vaccination was ultimately associated with an increased risk of hospitalization in dengue-naïve individuals, the risk appeared globally reduced during the first two years (RR 0.212) [23]. Specific data about the 2-year follow-up for the seronegative subgroup was not available, but the overall lower incidence of clinically severe dengue may have contributed to the delayed recognition of the increased hospitalization risk in seronegative individuals when assessed over longer timeframes. Bayesian analysis of the Asian and Latin American trials confirmed the higher risk of severe VCD and hospitalization in seronegative subjects [24].

Regarding immunogenicity, pooled data of 5780 participants from the Asia-Pacific region, South America and the U.S.A. showed that geometric mean titers (GMTs) of neutralizing antibodies increased from baseline to 28 days after the third dose against all four DENV serotypes across all geographical regions, age groups and serostatuses. Individuals who were seropositive for at least one serotype typically achieved higher GMTs than naïve participants; however, seronegative individuals generally exhibited a greater fold increase from baseline to post-third dose. Notably, GMTs were higher in individuals over 11 years old coming from endemic areas when compared to children and toddlers, though when only considering seronegative subjects, children developed higher GMTs than seronegative adolescents and adults [25]. This may be linked to the lower baseline seroprevalence in younger individuals, as preexisting flavivirus immunity increases vaccine immunogenicity [26,27,28]. The immune response against serotype 1 was generally lower compared to the other serotypes, with a GMT increase from baseline ranging from 1.42- to 9.99-fold for serotype 1, from 2.03- to 18.9-fold for serotype 2, from 2.46- to 19.8-fold for serotype 3; and from 3.35- to 22.9-fold for serotype 4. GMTs against all serotypes decreased within the first two years after administration regardless of the age group or serostatus, but remained above baseline throughout years 3 and 4 [25].

A pooled analysis of 18 clinical trials (evaluating a total of 77,234 doses of CYD-TDV and 36,006 doses of placebo) was also conducted to evaluate the safety of the CYD-TDV vaccine. Vaccine recipients showed a higher incidence of solicited local reactions compared to placebo, the most common being mild pain at the injection site (48% of participants receiving CYD-TDV against 37% of participants receiving placebo), generally resolving within 3 days. Solicited systemic reactions, including headache, malaise, and myalgia, were also more frequent among vaccine recipients (51%, 42%, and 40%, respectively, vs. 44%, 35%, and 33% for placebo). These reactions typically subsided within 3 days. The prevalence of solicited reactions was higher after the first dose. Unsolicited non-serious adverse reactions showed a statistically significant difference between the vaccine and placebo only in the 18–60 years age group. Injection-related reactions occurred in 4.6% of the participants receiving CYD-TDV and 1.6% of participants receiving placebo. The most common were hematoma (2.8% of participants receiving CYD-TDV against 0.8% of participants receiving placebo), gastrointestinal disorders (0.7% vs. 0.3%) and infections (0.7% vs. 0.2%). Unsolicited non-serious adverse reactions were generally mild, occurred within 3 days after injection and resolved within 3 days. The prevalence of non-serious unsolicited adverse reactions was also higher after the first dose. Serious adverse events (SAEs) did not differ significantly between participants receiving the vaccine and those receiving placebo. Vaccine-related SAEs included urticaria, asthma, acute polyneuropathy, polymyalgia rheumatica and headache. No deaths related to the CYD-TDV vaccine were reported. Post-vaccination low-level viremia was detected in 5.6% of tested individuals, with no effect on solicited and unsolicited adverse events. The participant’s serostatus did not affect the rates of solicited and unsolicited adverse events [28,29].

Some trials showed that reactogenicity and efficacy did not vary significantly when CYD-TDV was co-administered with other vaccines, such as the yellow fever vaccine or the combined tetanus toxoid, reduced diphtheria toxoid and acellular pertussis vaccine [30,31,32].

Although all trials followed a 0, 6 and 12-month injection schedule, trial NCT01943825 explored a shorter schedule of 0, 2 and 6 months, showing little difference in GMTs post-third dose. No difference was reported regarding SAEs and solicited adverse events; however, headache and malaise were more common with this shorter schedule. Another phase II trial demonstrated the non-inferiority of a two-dose schedule in terms of GMTs against each serotype at both 28 days and 1 year among seropositive individuals, suggesting that this schedule could be a valid alternative, potentially improving compliance and vaccine coverage [33]. Data on the actual efficacy against VCD and hospitalization following shorter schedules are still lacking.

Two phase II trials investigated the efficacy of CYD-TDV when given as a booster dose to individuals vaccinated 4–6 years prior. Both studies showed an increase in GMTs against all four serotypes comparable to post-dose 3 levels [34,35,36]. Again, actual clinical efficacy data for this practice have yet to be reported. Currently, the vaccine manufacturer (Sanofi Pasteur) officially discontinued vaccine production due to a significant drop in market demand [37,38].

### 3.2. TAK-003

TAK-003 is a live-attenuated tetravalent vaccine, with its backbone based on an attenuated laboratory-derived virus (TDV-2), obtained through culture of DENV-2 in primary dog kidney (PDK) cells over 53 passages, and the other three virus strains (TDV-1, TDV-3 and TDV4) generated by replacing the prM and E genes of TDV-2 with those from wild-type of DENV-1, DENV-3, and DENV-4 strains.

A first phase-2 clinical trial conducted on 1800 children aged 2 to 17 years living in endemic areas (Dominican Republic, Panama, and the Philippines) compared different administration schedules (single dose, vs. primary dose plus booster at day 365, vs. two doses at day 1 and 91, vs. placebo). A peak in GMTs was observed in seronegative individuals after the one-year booster dose; however, there were no significant differences in GMTs at 4 years between different dose schedules; notably, higher GMTs were observed in seronegative subjects when a two-dose schedule was administered. Results from this clinical trial supported the two-dose schedule over the one-dose schedule and the three-months schedule over the one-year-booster-schedule due to the potential additional protection during the first year after vaccination for seronegative subjects. While vaccine efficacy was not assessed in this study, a significantly lower risk of VCD was reported in the vaccine groups compared with placebo in the 1479 participants that completed study visits to the 4-year study period, with a 0.35 RR of VCD in TAK-003 recipients compared to placebo. RR was lower for DENV 2 (0.15) than DENV 1 (0.35) and DENV 3 (0.50). It was not possible to calculate RR for DENV 4 as no cases were reported in the placebo group [39,40]. This study did not evidence any safety concerns [41]. More specifically, including a high percentage of previously unexposed subjects, this study evidenced the long-term safety in this group in terms of severe dengue development. Considering the absence of any approved vaccine for dengue-naïve individuals, this was the first evidence of a promising vaccine filling the unmet clinical need of an effective and safe vaccine in this group [39].

The efficacy and safety of TAK-003 were further evaluated in a five-part phase III clinical trial involving 20,099 children and adolescents aged 4–16 years living in endemic areas of Asia and Latin America (vaccine:control ratio 2:1). At the 12-month follow-up (19,021 participants), VE was 80.9% against VCD and 95.4% against hospitalized dengue, with lower VE in the seronegative group (74.9%). The highest efficacy was observed against DENV-2 (97.7%) and DENV-1 (73.7%). VE was lower against DENV-3 (62.2% overall), and no efficacy was observed against DENV-3 in the dengue-naïve subgroup. Data were insufficient to evaluate overall VE against DENV-4 [41]. At 18 months, VE decreased slightly to 73.3% against VCD (66.2% in seronegative individuals, 76.1% in seropositives), 90.4% against hospitalization and 85.9 against hemorrhagic forms [42]. By the third year 18,987 participants completed the follow-up and cumulative VE further declined to 62.0% against VCD (54.3% in seronegatives, 65.0% in seropositive), though VE against hospitalization remained high at 83.6% (77.1% in seronegatives, 86.0% in seropositives). Data confirmed the highest VE against DENV-2, but vaccine was also effective against VCD for all serotypes in seropositive groups (56.2% for DENV-1, 83.4% for DENV-2, 52.3% for DENV-3, 60.7% for DENV-4). Efficacy was confirmed for DENV-1 (43.5%) and DENV-2 (91.9%) in seronegatives, but no efficacy was observed in DENV-3 seronegatives, whereas data were insufficient for DENV-4 seronegatives. What still needed to be clarified, due to lack of data, was whether inefficacy in preventing VCD in DENV-3 seronegative individuals would reflect on higher hospitalization rates. Of note, VE against hospitalized dengue were frankly imbalanced, as most hospitalizations were related to DENV-2, where VE was highest; this prevented meaningful subgroup analyses for hospitalization by serostatus and by serotype, but it suggests that TAK-003 provides great clinical benefit by preventing severe outcomes linked to the most virulent serotype [43].

Updated results at the end of part 3 of this five-part study were published in 2024, reporting results observed in the 18,257 participants who completed the 57 months follow-up after the first vaccination (or 4.5 years after the second dose). Cumulative VE against VCD was 61.2% (64.2% in the 14,517 dengue-exposed participants and 53.5% in the 5546 dengue-naïve participants). Efficacy against VCD was confirmed against all DENV serotypes (56.1% for DENV-1, 80.4% for DENV-2, 52.3% for DENV-3, and 70.6% for DENV-4) in seropositive participants, and against DENV-1 (45.4%) and DENV-2 (88.1%) in seronegative participants. Previous results regarding DENV-3 and DENV-4 in dengue-naïve participants were confirmed at this timepoint. Cumulative efficacy against hospitalized VCD persisted at 84.1% (85.9% and 79.3% in dengue-exposed and dengue-naïve participants, respectively). A summary of VE trends over time can be found in Table 1. The relatively long follow-up (4.5 years) of this study, from which no safety concerns emerged, also provided robust evidence of its long-term safety [44].

TAK-003 is the first vaccine to show promising long-term safety and efficacy in dengue-naïve individuals, a group previously without approved vaccine options. While VE is lower in this group compared to seropositive individuals, the vaccine still provides significant protection against hospitalization and severe disease, especially for DENV-1 and DENV-2. No efficacy against DENV-3 in seronegative individuals was observed at all timepoints. This is clinically important, as it may leave this subgroup vulnerable to DENV-3 infection, though the impact on hospitalization rates remains unclear due to insufficient data.

To address the trend of waning efficacy observed during years 2 to 3 in dengue naïve individuals, which could lead to an increased risk of ADE, the effect of a booster dose administered 4 years after the primary series will be assessed in the remaining part of the clinical trial. In this perspective, part 4 of the trial is ongoing, with the objective of evaluating safety and efficacy 13 months post-booster vaccination. Finally, part 5 will complete 2 years of follow-up after the booster dose.

Immunogenicity and safety of TAK-003 were also confirmed in a randomized clinical trial conducted in Mexico and involving 400 adolescents aged 12–17 years. This study confirmed tolerability and immunogenicity against all DENV serotypes (with higher GMTs for DENV-2) at 9 months after vaccination with two doses of TAK-003, administered three months apart. Tetravalent immunogenicity was observed in 99.6% and 85.8% at months 4 and 9, respectively. The most common adverse effects were mild or moderate, with pain at the injection site and headache being most frequently reported [45].

Despite evaluation of efficacy being more complicated in the adult population in endemic countries due to diffuse pre-exposition, exploratory immunological parameters have been studied in both children and adults, supporting a reasonable assumption about efficacy in adults.

A phase II RCT evaluated antibody titers, VCD and SAEs in 360 participants (249 vaccine; 111 control) at 36 months post-vaccination, including vaccinated subjects (vs. placebo) between 1.5 and 45 years. In this trial, efficient antibody responses were observed at three years post-vaccination, with seropositivity rates of 97.3%, 98.7%, 88.0% and 56.0% for DENV-1, -2, -3 and -4, respectively, compared with 48.9%, 44.4%, 46.7%, and 42.2% in the placebo recipients. At the 3-year follow-up, seropositivity rates varied significantly for DENV-4 according to serostatus at baseline (89.5% in seropositives versus 21.6% in seronegatives), whereas no differences in antibodies production against DENV-1/2/3 were observed between previously exposed and unexposed subjects. GMTs were also greater in vaccinated seropositive and seronegative subjects when compared to seropositive placebo recipients, except for the seronegative DENV-4 subgroup. Overall, 79% of vaccinated participants were seropositive at 4 months and 22% at month 36 in baseline seronegatives, vs. 94% and 89% in baseline seropositives, respectively. However, due to the low number of VCD over the study period, vaccine efficacy could not be evaluated in these terms [46].

Moreover, an open-label, single-arm phase 3 trial was conducted in the USA, to evaluate the immunogenicity and safety of TAK-003 in 200 adult participants with a follow-up period of 9 months. Despite the study being conducted in a non-endemic area, 14.1% of participants were DENV seropositive at baseline. In this study, seroconversion against all serotypes was present in >97% of participants at 120 days post-vaccination. These rates persisted at 9 months for DENV-1 and DENV-2, while seroprevalence decreased to 85.7% and 86.5% for DENV-3 and DENV-4, respectively. Most common solicited adverse events were headache (36.3%), myalgia (33.2%), malaise (24.9%), asthenia (20.2%) and fever (3.1%) [47].

### 3.3. TV003/TV005

Another dengue vaccine candidate is in the late stages of clinical development. TV003 and TV005 are two of five formulations of dengue vaccines developed by National Institute of Allergies and Infectious Diseases (NIAID), Bethesda, MD, USA. Their development began by selecting the most promising monovalent components and testing various tetravalent combinations for optimal efficacy. The final formulations include of four recombinant DENV serotypes. The DENV-1 and DENV-4 components (rDEN1Δ30 and rDEN4Δ30) introduced attenuating deletions in the 3′-untranslated regions of DENV-1 and DENV-4, respectively. Immunity against DENV-2 was achieved using a chimeric virus (rDEN2/4Δ30) that expresses the prM and E genes of DENV-2 inside the genetic backbone of rDEN4Δ30. For DENV-3, The rDEN3Δ30,31 strain required an additional 31-nucleotide deletion in addition to the 30-nucleotide deletion engineered in rDEN1Δ30 and rDEN4Δ30 [48,49,50]. In comparison to TV003, TV005 has an increased dosage of the DENV-2 component and was designed after early phase I trials suggested lower immunogenicity to DENV-2 with the TV003 formulation [51]. On subsequent studies, however, balanced production of tetravalent neutralizing homotypic antibodies was proved in the majority of individuals receiving either formulation [52,53,54]. Both vaccine formulations fulfilled all safety requirements up to 70-year-old subjects, including participants from USA, Thailand, Brazil and Bangladesh.

Encouraging results were obtained about TV003 protection against a DENV-2 challenge virus administered 6 months after vaccination, with 100% (21/21) of DENV-2 challenge virus recipients being protected against DENV viremia and clinical manifestations, and 100% of the 20 placebo recipients developing viremia, with 80% of rash manifestations and 20% of subjects developing neutropenia [55].

The Instituto Butantan in Brazil is currently completing a phase III trial using TV003, licensed under the name Butantan-DV, which began in 2016 recruiting 16,235 participants with a planned 5-years follow-up. As previous phase I and phase II trials did not show an increase in GMTs after a second dose of TV003 [51,56,57,58], the study protocol employed a single dose of Butantan-DV. The efficacy of a shorter TV003/Butantan-DV schedule was confirmed by one other phase II trial consisting of 300 participants [56]. Preliminary results about the 2-year follow-up for 10,215 vaccinated individuals and 5947 individuals receiving placebo have been published, showing a vaccine efficacy against symptomatic VCD of 79.6% in seronegative participants (i.e., 4855 individuals in the vaccine arm and 2700 in the control arm) and 89.2% in seropositive ones (5009 in the vaccine arm and 304 in the control arm). When considering age groups, the 2–6 years group (with a dengue seroprevalence of 17.7%) experienced a global vaccine efficacy of 80.1% (73.4% in seronegative participants), the 7–17 years group (61.2% seroprevalence) had a global vaccine efficacy of 77.8% (75.4% in seronegative participants) and the 18–59 years group (66.5% seroprevalence) saw a global vaccine efficacy of 90.0% (81.1% in seronegative participants). Serotype-specific efficacy was measured at 89.5% for DENV-1 and 69.6% for DENV-2. DENV-3 and DENV-4 were not observed during the follow-up period, preventing an assessment of vaccine efficacy against these serotypes. Of note, efficacy is lower than that reported in the previously described human infection model; the discrepancy is likely due to several factors, such as different viral injection pathway (needle vs. mosquito), repeated exposure, different circulating viral strains and uncontrolled timing between vaccination and infection. Non-serious solicited and unsolicited adverse events were more frequent in the vaccine group (58.3%) compared to the placebo group (45.6%). Most adverse events were mild to moderate in severity. The most common local solicited adverse event was pain at the injection site (14.9% of vaccine recipients), while the most common systemic events were headache (36.4%), exanthema (22.5%), asthenia (19.3%), generalized pruritus (18.9%) and myalgia (17.4%). SAEs did not differ significantly between the vaccine and placebo groups, and no deaths related to the injections were reported. Vaccine-related SAEs included Bell’s palsy, bronchospasm, facial paralysis, Guillain-Barré syndrome, peripheral neuropathy, transverse sinus thrombosis and viral infection [59]. Safety data for TV003 were comparable in other phase I and phase II trials [51,56,57,60].

While several ongoing studies are investigating TV005, two phase II trials (192 and 168 participants, respectively) have published results on this candidate vaccine so far. Rash was the most common solicited adverse event, occurring in 26–62% of vaccine recipients, followed by headache in 12–50%. Local solicited adverse events were reported in 1–5% of the participants receiving the vaccine. Elevated alanine aminotransferase levels were seen in 3% of the vaccinees. No SAEs related to the vaccine were observed. Following TV005 vaccination, most participants, including children, showed seropositivity for most serotypes, with 83% seropositivity to DENV-1, 99% to DENV-2, 96% to DENV-3, and 87% to DENV-4. In previously dengue-naïve subjects, seropositivity after the first dose was registered in 70–92%, 97–99%, 94–97% and 88–97% of participants receiving the vaccine, respectively. There was no significant increase in seroconversion following the second dose for any serotype. Overall, 96–98% achieved a trivalent or better response and 71–90% had a tetravalent response following the first dose of vaccine. All dengue-experienced vaccinees had a trivalent or better response, with 87% showing tetravalent response to vaccination. Among naïve participants, 93% developed an immune response for at least three serotypes, and 59% developed a tetravalent response. Post-vaccination GMTs were higher in the seropositive participants compared to seronegative ones [51,61].

Two randomized trials also assessed the efficacy of TV005 in protecting vaccine recipients against newly established DENV-2 and DENV-3 challenge viruses 6 months after vaccination. The two challenge viruses are derived from dengue outbreaks in Tonga and Indonesia, respectively, and due to their mild clinical impact on infected subjects, they are considered to be naturally attenuated DENV strains [62,63]. Results were published in 2024 and showed that TV005 was well tolerated and protected all vaccinated volunteers (44/44) from viremia and clinical symptoms (none infected in either group). Placebo recipients (41 in total) had 100% (21/21) and 85% (17/20) post-challenge viremia in the two studies, respectively, and all experienced rash following challenge with either serotype. While these results are promising, the attenuated nature of the challenge viruses may not fully replicate the virulence or transmission dynamics of wild-type DENV strains encountered in endemic settings. Therefore, while data strongly support TV005′s safety and immunogenicity in controlled human infection models, real-world efficacy against circulating DENV strains needs to be confirmed in field studies [64].

TV005 is currently being tested through a new human infection model clinical trial in Bangladesh, but no phase III clinical trial have been started so far [64].

### 3.4. TDEN

The Walter Reed Army Institute of Research (WRAIR), Silver Spring, MD, USA, and GlaxoSmithKline Biologicals (GSK), Rixensart, Belgium, developed a tetravalent DENV live-attenuated vaccine between 2003 and 2008 [65,66,67], which consisted of a combination of four lyophilized monovalent live-attenuated strains representing each DENV viral strain. Attenuation was achieved by culture in PDK cells over 53 passages. Two phase I/II trials for this vaccine initially proved safety and encouraging immunogenicity results of two vaccine doses, administered 6 months apart, in flavivirus-naive children and infants older than 12 months. Major concerns in these studies were: 1. detection of transient viremia in some vaccinated children, 2. waning immunity at one-year follow-up, exposing to an increased risk of ADE due to partial protection and 3. greater antibody responses against DENV-2 and DENV-4 than DENV-3 and DENV-1 [68]. To address these issues, a new rederived vaccine was developed (referred as TDEN) and its safety and efficacy were evaluated in flavivirus-naïve, but also DENV seropositive adults, in USA and Thailand with two RCTs. The first trial was conducted in USA in 2007 [69]; in this case, two different formulations of TDEN were administered (formulations F17 and F19) in two doses given six months apart (a third dose was administered after 5–12 months in some cases). Results showed that both the F17 and F19 formulations were safe and reactogenic compared to the older version, with reduced incidences of DENV-4 viremia. Of note, immunogenicity was considered as a surrogate outcome for clinical protection. In previously seronegative individuals, vaccine response was present in 37.5–40.0% of individuals after dose 1, reaching 60.0–66.7% after dose 2, with tetravalent seroconversion and slightly higher titers against DENV-2. A third dose administered 5 to 12 months after the second dose did not prove effective in increasing immunogenicity. Given the good clinical and laboratory tolerance after vaccination and the low incidence of DENV-4 viremia (5.3% of vaccine recipients), the authors theorized that the re-derivation and serial passage of the DENV-4 seed virus may have created a more attenuated DENV-4 strain, therefore supporting the acceptable safety profile of the new TDEN vaccine candidate. No safety concerns were detected based on this study results and the two new formulations were found to be comparable in terms of safety and immunogenicity.

Another phase II trial conducted in Thailand between 2007 and 2008 tested the F17 and F19 formulations in 120 healthy adults, most of whom had been previously exposed to flaviviruses (87.5% and 97.5% in the two vaccine groups and 92.5% in the placebo group). Participants received two doses six months apart, with a follow-up period of three months after the second dose. Both formulations proved safe, with no SAEs or dengue cases reported among vaccinated participants, whereas one case of DENV infection was reported in one placebo recipient. Low-level viremia was detected in a small number of participants (5 out of 80) post-vaccination, but these individuals were mostly asymptomatic or experienced mild symptoms like rash or fatigue. The vaccine induced strong tetravalent antibody responses in most participants, particularly those with pre-existing flavivirus immunity. One dose was sufficient to generate an immune response in previously exposed individuals, with 100% (in the F19 group) and 97.1% (in the F17 group) tetravalent antibody response 1 month after each vaccine dose (at pre-vaccination, 76.5% and 78.9% of subjects in F17 and F19 groups, respectively, were primed to all four DENV types). Among the six unprimed subjects, 5/6 were seropositive to each DENV type 3 months after the second dose; however, two doses were necessary to elicit a tetravalent immune response for four of these subjects. TDEN showed promising results in its ability to convert individuals with monovalent pre-vaccination antibody profiles into those with trivalent or tetravalent responses. This is significant because a monovalent response is associated with an increased risk of severe dengue in subsequent infections. Immune responses (measured by GMTs) to DENV-1 and DENV-2 serotypes seemed slightly superior to the immune responses observed for the DENV-3 and DENV-4 serotypes among the flavivirus-primed subjects. In the previous trial of the same vaccine formulations, this trend was also observed among flavivirus-unprimed subjects [70]. After proving safety and immunogenicity of the two formulations in two endemic (Thailand and Puerto Rico) and one non-endemic (USA) country, investigation on cell mediated immune response showed that both formulations of the live-attenuated TDEN vaccine candidate elicited only poor to moderate B-cell and T-cell responses, irrespective of the priming status of the participants. Moreover, the vaccine was shown to be unable to induce durable multivalent DENV-specific neutralizing antibodies responses [71]. Additional research would have been required to fully assess the protective effects of F17 and F19 vaccine candidates.

### 3.5. DPIV

DPIV is a tetravalent purified formalin-inactivated DENV vaccine, formulated with three different adjuvants (aluminum hydroxide or Adjuvant System (AS)01E or AS03B) at the dose of 1 μg or 4 μg of each DENV serotype, obtaining a total of four different vaccine formulations. When administered in two doses one month apart, it proved safe and immunogenic in two phase I clinical trials, conducted both in DENV-naïve and DENV-primed adults [72,73]. Safety and immunogenicity of all the DPIV formulations was later confirmed after 3 years of follow-up [74]. However, although production of well-balanced pick titers of tetravalent neutralizing antibodies was obtained, they decreased quickly after vaccine administration, especially in dengue-naïve subjects. Therefore, cell-mediated immune response after DPIV administration in dengue-naïve and dengue-primed adults was further characterized and results were published in 2022. Data showed that vaccine-induced memory B cells, specific to each DENV serotype, were higher in the dengue-primed than dengue-naive individuals. A subsequent DPIV booster dose induced strong anamnestic B-cell responses. Regarding T-cells, results showed production of multifunctional CD4+ T-cells in vaccine recipients, with higher production in previously seropositive subjects; moreover, CD4+ T-cells in vitro proliferative capacity and antigen-specific production of several inflammatory chemokines was reported. Both B-cells and T-cells response was demonstrated to be persistent up to 12 months, suggesting that specific immunity could persist effective despite the previously described waning of neutralizing antibodies over time. Investigators recognized that the phenomenon of waning neutralizing antibodies, despite parallel persistence of cellular immunity, needs further study in order to be better understood and characterized, and waning immunity should be ruled out before further development of the DPIV vaccine formulations, as insufficient neutralizing antibodies would be connected to the risk of ADE [75].

### 3.6. V180

V180 is a candidate recombinant subunit vaccine based on all four DENV strains envelope glycoprotein. Safety and immunogenicity of nine V180 formulations were studied in 98 flavivirus-naïve adults through a phase I clinical trial conducted in Australia and results were shared publicly in 2019. The different formulations included either ISCOMATRIX adjuvant (2 dosage levels), aluminum-hydroxide adjuvant, or were unadjuvanted, and were compared to phosphate-buffered saline placebo. Administration schedule was based on three injections at months 0, 1 and 2 and patients were followed-up until 1 year after last vaccine administration. Although all formulations proved safe, higher frequency of adverse events was observed in ISCOMATRIX adjuvant vaccine recipients. On the other hand, while all ISCOMATRIX adjuvant formulations showed robust immunogenicity, the aluminum-adjuvanted and unadjuvanted formulations were poorly immunogenic. Interestingly, immunogenicity did not increase depending on V180 or ISCOMATRIX dose, suggesting that just the presence of this adjuvant was effective in inducing strong and persistent immune response. Notably, despite increase in the amount of the DEN4-80E component compared to previous formulations, DENV-4 neutralizing antibodies measured by GMTs were generally lower and less durable than for the other serotypes, although the memory B-cell responses were balanced across all serotypes. Although induction of neutralizing antibodies could suggest protection against DENV infection, further studies are needed to prove effective clinical protection for this vaccine candidate. Moreover, the proportions of participants exhibiting a tetravalent antibody response declined over time. Authors concluded that, since partial and waning immunity represent a theoretical risk for post-vaccination enhancement of dengue disease, V180 as a stand-alone vaccine may not represent an optimal approach for protecting against dengue disease [76].

While NIAID’s TV003 and TV005 live-attenuated dengue vaccine (LATV) had proven safe and able to induce production of tetravalent neutralizing antibodies after a single primary dose [51,57], a question was still open about the necessity of a booster dose, and a second TV003 or TV005 dose had not proven efficient in increasing neutralizing antibodies titers in primed subjects [58]. Since the low immunogenicity of the boost dose was attributed to the inhibition of replication of the attenuated virus due to presence of high levels DENV neutralizing antibodies, it was hypothesized that this problem could be overcome through administration of a recombinant vaccine as a booster dose, the effect of which should be less affected by production of specific antibodies.

Therefore, in a subsequent study, safety and immunogenicity of a single booster dose of the V180 vaccine candidate were tested in 20 adults who had previously received 1 or 2 doses of LATV. V180 was administered either unadjuvated or adjuvated with Alhydrogel. The V180 booster doses were immunogenic and generally well tolerated in these dengue-experienced participants after a 6-month follow-up period. Administration of V180 increased DENV-specific serum neutralization titers at day 28 and at 6-month follow-up; moreover, seropositivity frequency increased in participants previously vaccinated with LATV. However, these levels failed to meet the protocol-specified criteria for a positive booster response and declined over time, although it is not known if reached levels could offer any protection against dengue infection or clinical benefit in case of infection. Further studies will be needed to answer these questions [77].

### 3.7. TVDV

Immunization against DENV through DNA components has been investigated due to their stability, non-replicating properties and manufacturing feasibility.

Supported by the Walter Reed National Military Medical Center (NMMC), Silver Spring, MD, USA, a tetravalent DNA vaccine (TVDV) was studied in a phase I clinical trial in dengue-seronegative healthy adults aged 18–45, of which results have been disseminated in 2018. It is based on prM and E protein coding sequences cloned in VR1012 plasmid and co-administered with Vaxfectin as an adjuvant.

Indeed, an earlier phase 1 clinical trial had showed the vaccine to be well tolerated and capable of generating adequate IFNγ T-cell responses, but poor anti-dengue neutralizing antibody responses [78], raising concerns about incomplete protection and breakthrough infections. Furthermore, as antibody-dependent enhancement is a major threat, non-specific antibody responses may increase the risk of severe disease upon subsequent heterologous infections. In this case, the vaccine candidate was administered through three intramuscular injections, at the doses of 1 to 2 mg, on day 0, 30 and 90, with and without the adjuvant Vaxfectin (1 mg). Dengue specific antibodies production and IFNγ production were assessed after each dose and monthly thereafter until day 270.

In terms of tolerability, no SAEs were reported related to vaccine administration and the vaccine proved safe and well tolerated. The most common adverse events included mild to moderate pain and tenderness at the injection site, typically resolving within 7 days. Fatigue, headache and myalgias were reported by the 42.5%, 45% and 47.5% of subjects, respectively.

Although the presence of Vaxfectin provided some benefits in producing higher neutralizing antibodies responses compared to TVDV alone, no sufficient antibodies production was present in most subjects. On the other hand, T-cell responses were reported in 70%, 50%, and 79% of subjects receiving 1 mg of TVDV alone, 1 mg of TVDV plus Vaxfectin or 2 mg of TVDV plus Vaxfectin, respectively; this dose-dependent increase in cellular response was not considered statistically significant. Currently, DENV vaccine efficacy is mainly determined based on the presence of neutralizing antibodies production, and there is no sufficient evidence about the effectiveness in protecting against dengue infection when anti-dengue cellular immunity alone is present. Since the clinical trial appeared to show dose-dependent increased immune responses, authors hypothesized that higher vaccine doses combined with Vaxfectin (4 mg DNA/1 mg Vaxfectin per injection) could be tested in future studies and likely improve humoral immune responses [79].

## 4. Discussion

DENV infection induces the production of lifelong protective, homotypic, neutralizing antibodies [80]. However, while heterotypic protection (i.e., against DENV serotypes that are different to the one that caused the primary infection) is usually present during the first years after infection, waning heterotypic immunity is usually observed over a few years, inducing higher risk of severe disease in case of infection with a new serotype [81]. Indeed, the production of non-neutralizing antibodies in the presence of a new DENV serotype induces a phenomenon known as antibody-dependent enhancement, which is the main mechanism favoring enhanced viral entry into the host’s cells and high infection burdens with consequent systemic vascular leak syndrome, potentially leading to shock and hemorrhagic and fatal manifestations [82].

Due to these immunologic mechanisms, an ideal vaccine should be protective against all DENV serotypes, in the absence of waning immunity phenomena. This is particularly important in case of dengue-naïve individuals, since partially protective immunization could mimic a first infection, therefore representing a risk of severe disease in case of a secondary infection. Moreover, since waning immunity could increase the risk of severe dengue, it is important that vaccines guarantee persistent specific immune response over time [83]. Based on these principles, vaccine development for DENV infection has been challenging [84], more so considering different VE and safety profiles in seronegative and seropositive individuals. Indeed, applying WHO seroprevalence thresholds [85] for mass vaccination in endemic countries poses some challenging problems: 1. the sensitivity and specificity of commonly available tests can be suboptimal, especially in areas where cross-reactivity with other flaviviruses may be common [86]; 2. dengue transmission is spatially heterogenous and achieving representative sampling across diverse geographic and socioeconomic strata is logistically complex and resource-intensive [86]; and 3. limited laboratory capacity, trained personnel, and funding prevent meaningful serosurveys and the use of gold-standard assays. Currently, two vaccines passed phase III trials and were licensed for their use in several countries, while other vaccine candidates are at different development stages, within a promising and variegated scenario, as illustrated in Table 2.

The first licensed DENV vaccine was the CYD-TDV vaccine (under the commercial name of Dengvaxia), approved by the European Medicines Agency (EMA) and Food and Drug Administration (FDA) in 2018 and 2019, respectively [87,88]. Its use is indicated in DENV seropositive individuals aged 9–45 years (or 9–60 years, depending on the country-specific regulatory approvals) living in dengue-endemic areas. Its efficacy against VCD has been proved to be 32% at 5 years among seronegative and 73% among seropositive individuals, being higher against DENV-3, and DENV-4 strains compared to DENV-1 and DENV-2. Importantly, an increased risk associated with vaccination of seronegative individuals, with higher hospitalization rates due to VCD, has been reported. Therefore, the WHO Strategic Advisory Panel recommended against vaccination for seronegative patients, as well as FDA and EMA approved vaccination only in documented seropositive individuals. WHO suggests that vaccination in the absence of documented seropositivity could be considered in countries where >80% population is seropositive by 9 years of age [89]. To date, the vaccine is not widely used, since measurement of DENV antibodies in all vaccine candidates is unpractical, especially in DENV endemic countries [90]. Moreover, the manufacturer stated that discontinuation of this vaccine has been decided due to a lack of demand in the global market [37,38].

TAK-003 was the second licensed dengue vaccine (under the commercial name of Qdenga). At 4.5 years after the second dose, cumulative VE against VCD and hospitalized dengue was 61.2% and 84.1%, respectively. Efficacy against VCD was confirmed against all DENV serotypes in seropositive participants, but only against DENV-1 and DENV-2 in seronegative participants. Demonstration of overall efficacy of TAK-003 in preventing VCD and hospitalized dengue independently of serostatus marked an important step, since these results encouraged the possibility of vaccination for seronegative individuals. Currently, the effect of a booster dose administered 4 years after the primary series is being assessed for TAK-003. Moreover, a post-authorization study is planned to assess VE against hospitalized dengue, with the aim of overcoming limitations to the evaluation of TAK-003 impact on hospitalized cases due to DENV-3 and DENV-4 [91].

**Figure 2:**
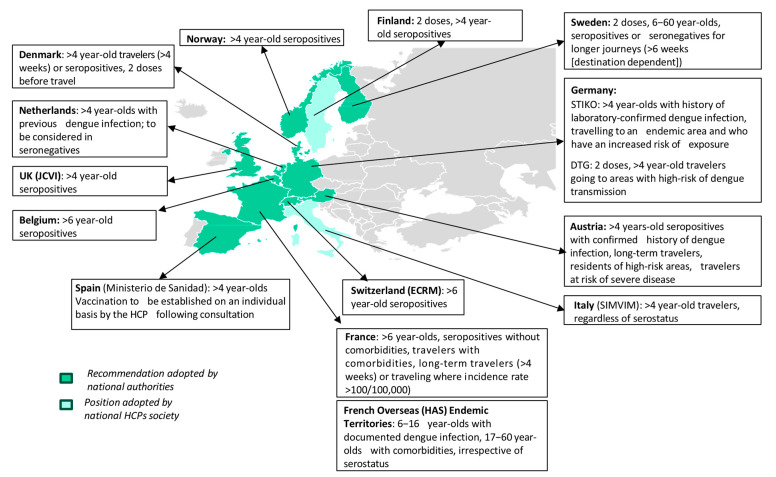
Currently available recommendations regarding dengue vaccine indications for travelers issued by national authorities and scientific societies in the European context [93,95,96,97,98,99,100,101,102,103,104,105,106,107,108].

WHO recommends vaccination with TAK-003 for children aged 6–16 years in endemic areas and supports the introduction of TAK-003 into routine immunization programs in these settings. However, due to lack of data demonstrating VE against DENV-3 and DENV-4 in seronegative individuals, WHO does not recommend the programmatic use of TAK-003 vaccine where dengue transmission is low or moderate. As regarding vaccination in the adult population, since TAK-003 efficacy was mainly studied in children and adolescents, WHO supports vaccination in adult subjects with comorbidities living in dengue-endemic countries, with an upper age limit of 60 years [90]. Indeed, evaluation of vaccine efficacy is challenging in the adult population, due to high seroprevalences in this population group. However, available data suggest that immunogenicity of TAK-003 in adults is comparable to the younger population, suggesting that comparable efficacy could be expected in older groups. The vaccine is approved for subjects ≥ 4 years by EMA, but age indications vary between different countries, mainly due to weak scientific evidence. According to May 2024 WHO’s position paper, travelers with previous history of dengue infection could also benefit from vaccination to prevent a second infection when traveling again to an endemic country. Frequent travelers, long-term travelers, migrants, and long-term expatriates, due to higher probability of being seropositive, could also benefit from vaccination. However, they should be informed that the highest benefit of vaccination is when epidemics due to DENV-2 or DENV-1 are ongoing at the destination, whereas the vaccine may not protect against DENV-3 and DENV-4 if they are seronegative, with a potential risk of severe dengue in case of exposition to DENV-3 and DENV-4. In certain cases, pre-vaccination serological screening could help balancing risks and benefits of TAK-003 vaccination in travelers [89]. As regarding the field of travel medicine, in the wake of the WHO recommendations, a number of scientific societies and national authorities have issued heterogeneous country-specific indications [92,93,94,95,96,97,98,99,100,101,102,103,104,105,106,107,108]. A summary of currently available recommendations regarding dengue vaccine indications for travelers issued by national authorities and scientific societies in the European context is represented in Figure 2. Due to a lack of published data, each position regarding vaccine administration to different population groups and in different epidemiological settings should be taken with caution until stronger evidence is available on this field. As regards FDA approval for TAK-003, the pharmaceutical company withdrew the application for its approval in the United States in July 2023 due to lack of data required by the FDA for the vaccine commercialization [109]. WHO, FDA and EMA recommendations on CYD-TDV and TAK-003 vaccines are summarized in Table 3.

In addition to the successful development of the two licensed live-attenuated vaccines, a third live-attenuated vaccine, administered in a single dose schedule, and developed by NIAID, is in late-stage development. So far, promising preliminary results about efficacy of the TV003 and TV005 vaccine candidates have been reported in different age groups (including adults and children). Simpler dosing schedule and well-balanced tetravalent immune response make it a strong candidate for broad use in the upcoming future. Currently, a phase III clinical trial using TV003 (licensed under the name Butantan-DV) is ongoing in Brazil, which began in 2016 with a planned 5-years follow-up. TV005 is currently being tested through a human infection model clinical trial in Bangladesh, but no phase III clinical trial have been stared so far. Of note, studies on TV003 and TV005 brought new light on the role of controlled human infection models to test DENV vaccine candidate efficacy. Indeed, although with some limits such as the inapplicability of the model for all possible viral strains, the inability to test protection duration and to extend the trial to larger population samples, they offer advantages in deepening the knowledge about efficacy and safety of new vaccine candidates, as well as viral infection and replication processes, especially when dealing with serotypes that are not commonly circulating in specific areas where clinical trials are conducted.

In addition to the advanced-stage candidate vaccines, newer vaccine approaches are under development. While traditional vaccine platforms (i.e., live-attenuated, inactivated and recombinant subunit vaccines) have established safety and manufacturing processes, suboptimal immunity in seronegative individuals and/or the risk of ADE could steer the direction of future research towards emerging technologies. DNA vaccines already proved to be stable, economically acceptable, and conveniently producible; however, low immunogenicity limits their use. Newer strategies are also under investigation, such as the development of mRNA vaccines and nanoparticle-based vaccines, NS1-based vaccines targeting nonstructural proteins and immunization against specific targets that could interrupt the life cycle of DENV after the mosquito bite [84]. mRNA vaccines have demonstrated the ability to induce strong, balanced humoral and cellular immunity, with recent preclinical and early clinical data showing high efficacy and reduced ADE risk through epitope engineering [110]. Nanoparticle-based delivery systems enhance antigen presentation, stability, and immunogenicity, and can be tailored for multi-epitope peptide-based vaccines, potentially improving safety and efficacy profiles [111]. NS1-based vaccines and strategies targeting mosquito-stage antigens represent even newer approaches that may complement or be integrated with advanced platforms to overcome current limitations [84].

## 5. Conclusions

Development of an ideal DENV vaccine remains a challenge, since it should be effective against all DENV serotypes, approved for all age groups independently of serostatus, and safe, especially in terms of risk of developing severe dengue in subsequent infections. Moreover, despite older age and chronic diseases being independent risk factors for dengue mortality, most dengue vaccine candidates are being tested in younger and healthy population groups [89,90]. The evaluation of safety and efficacy in these categories should be among the primary objectives of vaccine trials in the next future, given the aging of the general population and increase in chronic illnesses worldwide. Analogously, immunocompromising conditions are increasing worldwide, but currently approved vaccines are contraindicated in immunocompromised subjects, who could instead benefit of some protection against DENV infection. This point will also be of paramount importance in the upcoming future.

Undoubtfully, also non-vaccine strategies are pivotal for the control of DENV transmission and spread, as the increasing number of cases is closely related to climatic changes, international travel and trade, interactions between humans, animals and the environment. For this reason, an appropriate pre-travel consultation is mandatory and new strategies need to be developed for vector control, considering connections between the disease epidemiology and climatic and environmental factors.

## Figures and Tables

**Table 1:** VE of TAK-003 against VCD and hospitalized dengue over a 4.5-year period [41,42,43,44].

Timepoint	VE vs. VCD (Overall)	VE vs. VCD (Seronegative)	VE vs. VCD (Seropositive)	VE vs. Hospitalized Dengue (Overall)	VE vs. Hospitalized Dengue (Seronegative)	VE vs. Hospitalized Dengue (Seropositive)
12 months	Overall: 80.9% DENV-1: 73.7% DENV-2: 97.7% DENV-3: 62.2% DENV-4: insufficient data	74.9% *	82.2%	95.4%	97.2%	94.4%
18 months	Overall: 73.3% DENV-1: 69.8%, DENV-2: 95.1% DENV-3: 48.9% DENV-4: insufficient data	66.2%	76.1%	90.4%	89.5%	89.7%
3 years	Overall: 62.0% DENV-1: 56.2%, DENV-2: 83.4% DENV-3: 52.3% DENV-4: 60.7%	Overall: 54.3% DENV-1: 43.5%, DENV-2: 91.9% DENV-3: insufficient data DENV-4: insufficient data	Overall: 65.0% DENV-1: 56.2%, DENV-2: 83.4% DENV-3: 52.3% DENV-4: 60.7%	83.6%	Overall: 77.1% DENV-1: 77.2%, DENV-2: non estimable DENV-3: insufficient data DENV-4: non estimable	Overall: 86.0% DENV-1: 69.2%, DENV-2: 95.3% DENV-3: 72.1% DENV-4: non estimable
4.5 years	61.2%	Overall: 53.5% DENV-1: 45.4%, DENV-2: 88.1% DENV-3: insufficient data DENV-4: insufficient data	Overall: 64.2% DENV-1: 56.1% DENV-2: 80.4% DENV-3: 52.3% DENV-4: 70.6%	84.1%	Overall: 79.3% DENV-1: 78.4% DENV-2: non estimable DENV-3: insufficient data DENV-4: non estimable	Overall: 85.9% DENV-1: 66.8% DENV-2: 95.8% DENV-3: 74.0% DENV-4: non estimable

* No efficacy vs. DENV-3 in seronegatives.

**Table 2:** Main characteristics of vaccines and/or vaccine candidates that are licensed or in late-stage development.

Vaccine	Type of Vaccine	Producer	Development	Reference
CYD-TDV	Recombinant live-attenuated tetravalent vaccine, based on substitution of the yellow fever vaccine (YF17D) prM/E genes, with the genes of each dengue serotype	Sanofi Pasteur	Licensed (manufacturing suspended)	[23]
TAK-003	Live-attenuated tetravalent vaccine, backbone based on DENV-2 primary dog kidney (PDK)–53, and substitution of the pre-membrane and envelope genes of TDV-2 with those from wild-type DENV-1, DENV-1, DENV-3, and DENV-4 strains	Takeda	Licensed	[38]
TV003-TV005	Live-attenuated vaccine, obtained by the deletion of nucleotides from the 3′-untraslated regions of the DENV viral genome	National Institute of Allergy and Infectious Diseases (NIAID)	Phase IIIb	[53,58]
TDEN	Tetravalent live-attenuated DENV vaccine, made of four lyophilized monovalent live-attenuated strains representing each DENV viral strain	Walter Reed Army Institute of Research (WRAIR) and GlaxoSmithKline (GSK)	Phase II	[65]
DPIV	Tetravalent inactivated DENV vaccine, with three different adjuvants (aluminum hydroxide or AS01E or AS03B)	WRAIR, GSK and Fiocruz (Rio de Janeiro, Brazil)	Phase I	[69]
V180	Recombinant subunit vaccine based on all four DENV strains envelope glycoprotein	Merck & Co. (Rahway, NJ, USA)	Phase I	[70,71]
TVDV	Tetravalent DNA vaccine based on prM and E protein coding sequences cloned in VR1012 plasmid (co-administered with Vaxfectin as an adjuvant)	U.S. Army Medical Research and Development Command (Fort Detrick, MD, USA), WRAIR, NMRC and Vical (San Diego, CA, USA)	Phase I	[73]

**Table 3:** Indications released by EMA, FDA and WHO for the use of CYD-TDV and TAK-003.

Vaccine	EMA	FDA	WHO
CYD-TDV	Individuals 6–45 years with laboratory-confirmed previous dengue infection	People aged 9–16 years living in endemic areas, who have laboratory-confirmed previous dengue infection	Individuals aged 9–45 years or 9–60 years (depending on the country-specific regulatory approvals) living in dengue-endemic areas, who have laboratory-confirmed previous dengue infection
TAK-003	Adults, adolescents and children from 4 years of age.	Not licensed by FDA	Children aged 6–16 years in high-transmission settings. Persons with comorbidities in dengue-endemic countries could be offered vaccination, even if they fall outside the recommended age range, with the upper limit of 60 years.

## Data Availability

No new data were created or analyzed in this study. Data sharing is not applicable to this article.

## Supplementary Materials

The following supporting information can be downloaded at: https://www.mdpi.com/article/10.3390/idr17050117/s1.

## Abbreviations

The following abbreviations are used in this manuscript:

ADE	Antibody-dependent enhancement
CYD-TDV	Chimeric yellow fever virus—DENV-tetravalent dengue vaccine
DENV	Dengue virus
GMTs	Geometric mean titres
LATV	Live-attenuated tetravalent vaccine
RR	Relative risk
SAEs	Serious adverse events
VCD	Virologically confirmed dengue
VE	Vaccine efficacy
